# A Pilot Study on the Role of TRAFs in the Development of SARS-CoV-2 Infection Before and After Immunization with AstraZeneca Chadox1 in Mice

**DOI:** 10.3390/cimb47030165

**Published:** 2025-02-28

**Authors:** Mounia Ammara, Inass Samiry, Younes Zaid, Mounia Oudghiri, Abdallah Naya

**Affiliations:** 1 Immunology and Biodiversity Laboratory, Department of Biology, Faculty of Sciences Ain Chock, Hassan II University, Casablanca 20000, Morocco; ammara.mounia18@gmail.com (M.A.); inass.samiry2015@gmail.com (I.S.); younes_zaid@yahoo.ca (Y.Z.); mouniaoudghiri@gmail.com (M.O.); 2 Materials, Nanotechnologies and Environment Laboratory, Department of Biology, Faculty of Sciences, Mohammed V University, Rabat 10000, Morocco

**Keywords:** SARS-CoV-2 infection, CHADOX1 vaccine, platelet transcriptomics, TRAF receptors

## Abstract

The TRAF family of molecules are intracellular signaling adaptors that regulate various signaling pathways. These pathways are not only mediated by the TNFR superfamily and the Toll-like receptor/IL-1 receptor superfamily but also by unconventional cytokine receptors like IL-6 and IL-17 receptors. Overactive immune responses caused by TRAF signaling following the activation of these receptors frequently result in inflammatory and autoimmune diseases such as rheumatoid arthritis, inflammatory bowel disease, psoriasis, and autoinflammatory syndromes. Therefore, it is crucial to comprehend the signaling processes controlled by TRAFs, which have a significant influence on the determination of cell fate (life or death) and the functioning, specialization, and endurance of cells in the innate and adaptive immune systems. Our data indicate that the dysregulation of cellular expression and/or signaling of TRAFs leads to the excessive production of pro-inflammatory cytokines, hence promoting abnormal activation of immune cells. The objective of our investigation was to comprehend the function of these molecules in SARS-CoV-2 infection both prior to and during SARS-CoV-2 vaccination. Our results demonstrate a clear inactivation of the TRAF5 and TRAF6 genes when infection occurs after immunization, in contrast to infection without prior vaccination. This can bolster the belief that immunization is essential while also demonstrating the involvement of these molecules in the pathogenesis of SARS-CoV-2.

## 1. Introduction

The COVID-19 pandemic, caused by the novel coronavirus SARS-CoV-2, has underscored the critical need to understand the complex interplay between viral pathogenesis and host immune responses. While much attention has focused on the role of immune cells and cytokines in COVID-19, emerging evidence highlights the importance of platelets as key mediators of inflammation, thrombosis, and immune modulation during viral infections [1,2]. Platelets are no longer viewed solely as hemostatic cells; they actively participate in the immune response by interacting with viruses, releasing inflammatory mediators, and forming platelet–leukocyte aggregates that amplify tissue inflammation and contribute to disease progression [1]. In the context of SARS-CoV-2 infection, platelets have been implicated in the thromboinflammatory complications that characterize severe COVID-19, including deep vein thrombosis, pulmonary embolism, and multi-organ dysfunction [2]. A key study by Zaid et al. demonstrated that platelets can associate with SARS-CoV-2 RNA, leading to their hyperactivation in COVID-19 patients [3]. However, the molecular mechanisms underlying platelet activation and their contribution to COVID-19 pathogenesis remain poorly understood.

Among the molecular players involved in immune regulation and inflammation, tumor necrosis factor receptor-associated factors (TRAFs) [4] have emerged as critical modulators of cellular responses. These signaling molecules not only govern immune cell survival and function but also interact with various receptor systems, including those on platelets, further underscoring their potential role in COVID-19 pathogenesis. For example, they regulate the growth of T cells, modulate the functionality of macrophages, and influence the survival decisions of B cells. TRAFs can function as adaptors, E3 ubiquitin ligases, or both simultaneously. As adaptors, they make it easier for receptors and cellular mediators to send and receive signals. As E3 ubiquitin ligases, they control the breakdown of proteins and the starting of signaling cascades. It operates by acting on several receptors, including TNFRs, TLRs, interleukin, interferon, antigen, NOD-like, RIG-I, platelet, and transforming growth factor-β receptors [5,6].

SARS-CoV-2 induces and mediates the activation of the NF-kB signaling pathway during the SARS-CoV-2 infection through RIP/TRAF-dependent and RIP/TRAF-independent pathways [7]. The RIP/TRAF-dependent pathways and the RIP-dependent/TRAF-independent IKKβ activation pathways are interconnected routes that collectively lead to the activation of NF-κB signaling. The activation of IKKβ, which may involve interaction via the antigen receptor, suggests to IKK that it is further along in the process. A number of kinases implicated in NF-κB signaling pathways signal to IKK after RIPs and TRAFs. Specific kinases such as TAK1 are key participants in NF-κB signaling pathways and are associated with RIPs and TRAFs in mediating cellular responses. To be more specific, TAK1 mediates the TNF-alpha and IL-1 signaling pathways by activating IKK. Specifically, TAK1 mediates the inhibition of RIP/TRAP in TNF-alpha and IL-1 signaling pathways. Ligands (TNF-alpha, IL-1, and SARS-CoV-2 spike proteins) activate RIP/TRAP-dependent pathways, which include the TNF receptor 1, IL-1 receptor, and TLR4 receptor signaling pathways, respectively. Platelets are very involved in the pathogenesis of SARS-CoV-2 infection [8]; however, the complete mechanism has not yet been fully elucidated. What is certain is that platelets play a major role in the severity of cases of this disease [9]. The inflammatory nature of SARS-CoV-2 infection has prompted a focus on small molecules due to their role in various immune-mediated inflammatory diseases. Understanding the involvement of these molecules in the immune response could offer valuable insights for developing treatments for COVID-19. Understanding the role of these small molecules in the immune response could provide valuable insights into potential treatments for COVID-19. 

Given the importance of platelets in COVID-19 and the central role of TRAF proteins in immune signaling, we hypothesized that TRAF expression in platelets may be modulated during SARS-CoV-2 infection and vaccination, contributing to disease pathogenesis and vaccine-induced immunity. To test this hypothesis, we performed platelet transcriptomic analysis in a murine model of SARS-CoV-2 infection and vaccination. We focused on platelets because of their unique ability to integrate immune and hemostatic responses, making them a promising target for understanding the thromboinflammatory complications of COVID-19. By focusing on platelet TRAF signaling, our work bridges a critical gap in the understanding of COVID-19 pathophysiology. Platelets are uniquely positioned at the intersection of immunity and hemostasis, and their activation during SARS-CoV-2 infection may contribute to both the inflammatory and thrombotic complications of COVID-19. Our findings not only advance the understanding of platelet biology in viral infections but also provide a foundation for future studies exploring TRAF-targeted therapies for COVID-19 and other thromboinflammatory diseases.

## 2. Materials and Methods

Mice infection: Male and female K18-ACE2 mice, aged nine weeks, were purchased from the Jackson Laboratories (Bar Harbor, ME, USA). Mice were divided into four experimental groups: MOCK (n = 2), VAC (n = 3), VAC+INF (n = 3), and INF (n = 2). The MOCK group refers to control mice that underwent the same experimental procedures but were administered PBS solution instead of SARS-CoV-2. The infection was conducted using the (SARS-CoV-2 strain LSPQ, B1 lineage). Mice in the vaccinated groups received their immunization prior to infection, with SARS-CoV-2 exposure occurring 21 days post-vaccination. To assess the impact of infection and vaccination on platelet function, samples were collected 10 days post-infection.

Mice vaccination: The mice received an injection of 0.1 mL of the AstraZeneca CHADOX1 vaccine, which consists of at least 5 × 10^6^ chimpanzee adenovirus units of infectivity (U.I.) that express the SARS-CoV-2 Spike glycoprotein (ChAdOx1-S).

Mice platelets preparation: Platelets were obtained from mice at room temperature using approved techniques and standards established by the Ethics Committee of the Faculty of Sciences, Rabat, Morocco (Project: CEFSR/PR/2023-PR09). Concisely, blood was obtained by heart puncture, treated with acid-citrate-dextrose to prevent clotting, and mixed with warm PIPES saline glucose solution containing 300 nM of prostaglandin E1 (PSG/PGE). The blood was subjected to centrifugation at a force of 115× *g* for 10 min to obtain platelet-rich plasma. This plasma was then further diluted with PSG/PGE. Next, the platelet-rich plasma underwent centrifugation at 500× *g* for 10 min. The resulting platelet pellets were washed and then mixed with a modified Tyrode buffer solution. Following the depletion step, the purified platelets were rinsed in PSG/PGE prior to further processing.

RNA extraction and sequencing: The RNA-seq samples (mice platelets) underwent purification of total RNA from Trizol-treated samples, followed by treatment with DNase. The RNA-seq libraries were combined and analyzed using 75 base-pair, paired-end sequencing on an Illumina HiSeq-2000 machine (Illumina, San Diego, CA, USA). Each sample was assigned to a dedicated flow cell. The demultiplexing of sequencing data was performed, followed by conversion to FASTQ format. The alignment of paired-end reads to RefSeq was performed using TopHat2, and the calculation of RNA reads-per-kilobase-per million mapped (RPKM) was performed using RSeQC v2.3.9 (Boston, MA, USA).

RNAseq analysis using ExpressAnalyst: The RNA-seq count table was introduced to the web-based platform ExpressAnalyst (available at www.expressanalyst.ca, accessed on 18 October 2024) and we used the DESeq2 package to a complete transcriptomics and proteomics data analysis [10]. The differential expression analysis (DEA) and figures were obtained using the step-by-step thorough technique provided by Ewald et al. in the current protocols [11].

## 3. Results

Our results have been presented in many formats to enhance the clarity and understanding of their significance. The first figure below displays the results of comparing two groups: infected mice without prior vaccination (labeled as INF) and infected mice after vaccination (labeled as VAC+INF). The figures include both the control group and the vaccinated group, with the latter serving as a positive control in this case. 

**Figure 1:**
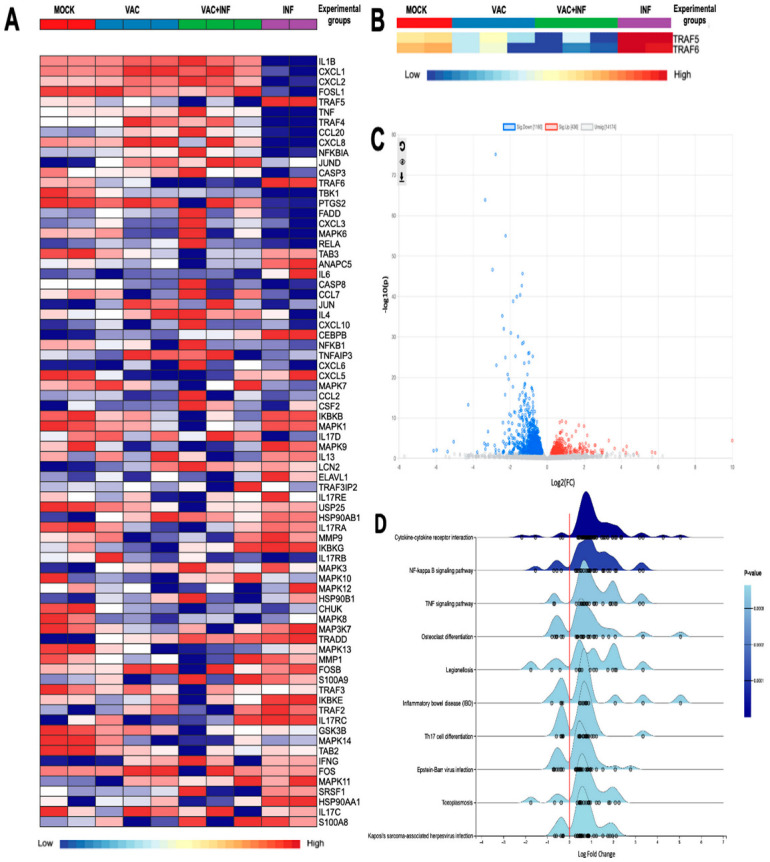
**The differentially expressed genes (DEGs) among the groups:** (**A**) Differential gene expression concerning platelet activation illustrated using a heatmap with transcript clustering using Ward’s method and multilevel and log (2) fold change analysis parameters. Normalized gene expression is represented between dark red for higher expression and dark blue for lower expression than the average. For the diagnosis bar: Mock is represented by a red color, vaccinated mice by blue, vaccinated then infected mice by green, and infected without prior vaccination by purple. (**B**) A customized heatmap highlighting only TRAF5 and TRAF6. (**C**) Volcano plot of differential gene expression analysis using DESeq2. Blue dots indicate 1160 downregulated genes; red dots 436 upregulated genes. Dots in light gray represent genes without significant regulation. (**D**) Ridge plot of enriched pathways based on differentially expressed genes. This figure illustrates the distribution of log2 fold change values for differentially expressed genes associated with various biological pathways. The *x*-axis represents the log2 fold change, while the *y*-axis lists the pathways, including cytokine–cytokine receptor interaction, NF-kappa B signaling pathway, TNF signaling pathway, and others. The density curves indicate the distribution of gene expression changes within each pathway, with individual dots representing specific genes. The color intensity corresponds to the *p*-value, with darker blue shades indicating higher statistical significance. The utilization of the enrichment network gene set facilitated the interpretation of the differential gene expression results acquired from RNA-seq. Additionally, it enabled us to categorize genes that are functionally related, thereby streamlining the analysis by emphasizing the most pertinent biological processes and metabolic pathways.

Figure 1A largely depicts the genes that we found interesting to investigate their expression within the context of our research. These genes have a direct implication in the immune response to SARS-CoV-2 infection and vaccination. We have proposed that the potential of the virus to cause disease may be related to inappropriate or imbalanced host immune responses. TRAF proteins have well-described roles in differentially regulating the expression of pro-inflammatory and antiviral interferon responses following the pattern recognition of viral antigens [12,13]. The software identified a total of 72 genes that showed differential expression when comparing the INF group to the VAC+INF group. These genes had a log2 fold change of 2.0 and a *p*-value equal to or less than 0.05. Our study specifically examined the TRAF family, as shown in Figure 1B. We observed a notable disparity in expression, with the TRAF5 and TRAF6 genes being significantly overexpressed in the INF group while being underexpressed in the VAC+INF group. In addition, we attempted to illustrate our findings using a volcano plot (Figure 1C), which offers a more effective way to visualize the variations in gene expression for both the genes of interest and all other genes derived from the RNAseq data. The genes play a role in multiple biological processes and signaling pathways, all of which are depicted on the ridge line chart (Figure 1D).

**Figure 2:**
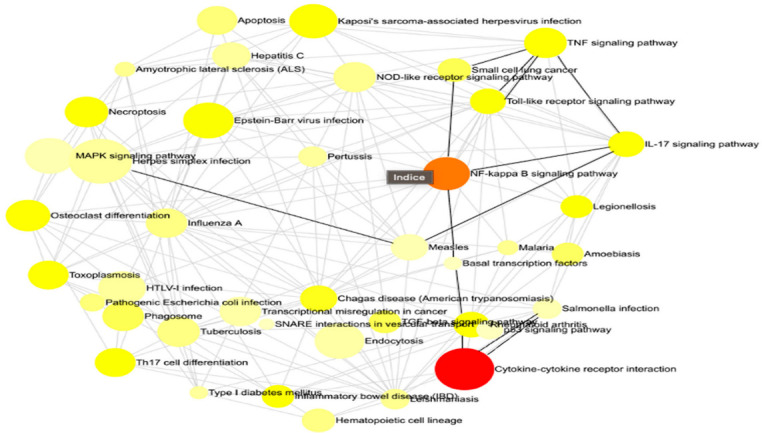
**The interaction net of the significant pathways.** Each node corresponds to a pathway, with its size indicating the degree of connectivity, meaning the number of other pathways it interacts with. Larger nodes represent pathways that share more genes with others, suggesting a central role in the biological response. The edges connecting the nodes indicate functional interactions, reflecting common genes involved in multiple pathways. The color of each node conveys the regulation status of the pathway, with red representing upregulated pathways, orange indicating downregulated pathways, and yellow signifying pathways containing both up- and downregulated genes. This network visualization provides insights into the global organization of biological responses and highlights key regulatory hubs that may play a crucial role in the studied condition.

This methodology enabled us to discern the principal governing networks influenced by our investigation, offering a clear and comprehensible representation of the intricate interconnections among various gene sets (Figure 2). Concisely, he emphasized all the pathways that were impacted throughout the infection both prior to and following immunization. We reference the statistically most significant pathways in our situation, namely, cytokine–cytokine receptor interaction, NF-kappa B signaling route, and TNF signaling pathway. 

## 4. Discussion

Platelets are increasingly recognized as key players in the immune response to viral infections, including SARS-CoV-2. Beyond their traditional role in hemostasis, platelets interact with viruses, modulate inflammatory responses, and contribute to tissue repair [1,14]. The TNF receptor-associated factor (TRAF) family of proteins, which regulate immune signaling pathways, may play a critical role in mediating these platelet functions during SARS-CoV-2 infection. Our study, which focused on platelet transcriptomics, reveals that TRAF expression is modulated in response to SARS-CoV-2 infection and vaccination, suggesting a potential link between platelet TRAF signaling and COVID-19 pathogenesis.

Platelets are equipped with a wide array of receptors and signaling molecules that enable them to respond to viral infections. For example, platelets express Toll-like receptors (TLRs) and cytokine receptors, which activate the downstream signaling pathways involving TRAF proteins [15]. Upon activation, platelets release inflammatory mediators (e.g., IL-1β, PF4, and RANTES) and form platelet–leukocyte aggregates, amplifying immune responses and contributing to tissue inflammation [1]. In the context of SARS-CoV-2, platelets may interact directly with the virus or respond to systemic inflammation, leading to dysregulated immune signaling and thrombotic complications [2]. Our findings suggest that TRAF molecules in platelets may mediate these processes, as evidenced by the differential expression of TRAF1, TRAF2, TRAF4, TRAF5, and TRAF6 in infected and vaccinated mice.

TRAF proteins are critical adaptors in immune signaling pathways, and their expression in platelets may influence the host response to SARS-CoV-2. For instance, TRAF6 is a key mediator of NF-κB and MAPK signaling, which regulate the production of pro-inflammatory cytokines [16]. In platelets, TRAF6 signaling may amplify the release of inflammatory mediators, contributing to the cytokine storm observed in severe COVID-19. Similarly, TRAF2 and TRAF5 are involved in TNFR signaling, which can modulate platelet survival and apoptosis [17]. Dysregulated TNFR signaling in platelets may exacerbate tissue damage and thrombotic events in COVID-19. Additionally, TRAF3 plays a role in type I interferon (IFN) production, which is essential for antiviral defense. SARS-CoV-2 has been shown to suppress IFN responses [18], and impaired TRAF3 signaling in platelets may further compromise viral clearance. Our data show that TRAF1 and TRAF4 are significantly upregulated in platelets from vaccinated and infected mice, suggesting that these molecules may play a role in modulating platelet responses to SARS-CoV-2. While the exact mechanisms remain to be elucidated, these findings highlight the potential importance of platelet TRAF signaling in COVID-19.

The exploration of the TRAF family [5], particularly TRAF5 and TRAF6, has garnered significant attention within immunological research, especially in the context of viral infections and vaccination responses [19,20]. In the foundational work by H. Park, 2018, the author elucidates the structural dynamics of the TRAF family, providing critical insights into receptor recognition and the functional implications of TRAF proteins in immune signaling pathways. Our research highlights underscore the importance of understanding these mechanisms, as they may contribute to the development of targeted therapies aimed at enhancing the efficacy of vaccines. Furthermore, the implications of these findings extend beyond the immediate context of SARS-CoV-2, potentially informing future studies on other viral infections.

However, the TRAFs family of receptors is not well researched in the context of platelet function, unlike in T cells, for example. Initially, TRAF5 expressed by naive CD4+ T cells, inhibits IL-6R signaling. The TRAF-C domain of TRAF5 regularly binds to gp130 (Il6st) in the cytoplasm. This region is between the first two of four phosphorylated tyrosine motifs that activate STAT3. TRAF5 and gp130 restrict JAK1, gp130, and STAT3 phosphorylation by IL-6 and soluble IL-6R. Similar inhibition is seen in TRAF2, which interacts with the same region [21]. TRAF5 suppresses initial IL-6R signaling in naive CD4+ T cells and enhances Th17 cell development in its absence. Retrovirus-induced TRAF2 inhibition in TRAF5−/− CD4+ T cell differentiation promotes Th17 cell development. In recipient mice with normal TRAF5 genes, donor CD4+ T cells lack the gene to aggravate EAE symptoms. The results show that TRAF5 and TRAF2 coupled to gp130 inhibit the IL-6 trans-signaling-activated JAK-STAT signaling pathway [22]. Additionally, The pro-inflammatory function of mature pDCs is negatively impacted by TRAF5 expression [23,24]. Moreover, TRAF5−/− mice have faster epidermal wound healing, a gradual inflow of pDCs, and increased wound-repair responses.

Furthermore, the cooperative interaction between the TRAF6- and TRAF2/3/5-binding sites in CD40 has been shown to increase the differentiation of B cells that produce antibodies [25]. The CD40-TRAF6 macrophage signaling pathway plays a major role in EAE progression. A chimeric Cd40 transgene with mutations at the TRAF6- and TRAF2/3/5-binding site controlled by the MHC-II promoter (MHCII-CD40-T6−/− and MHCII-CD40-T2/3/5−/−, respectively) reduces clinical scores of EAE and central nervous system demyelination in Cd40−/− mice. The cerebellum of MHCII-CD40-T6−/− mice has fewer immune cells and lower expression of pro-inflammatory genes such as Ifng, Il17, Tnf, Il6, and Mcp1. This study reveals that the CD40 signaling pathway, enhanced by TRAF6 in macrophages, affects EAE progression more [26]. 

Therefore, the single nucleotide polymorphisms (SNPs) of TRAF5 on Chromosome 1 have been linked to autoimmune disorders, specifically rheumatoid arthritis (RA) and uveitis [27]. According to a case-control study, hypermethylation of the CpG island in the TRAF5 gene promoter may cause AS. SLE patients had more B cells with low IgD, CD27, CXCR5, and CD21. This subpopulation of B-cells has reduced TRAF5 expression and increased TLR7 reactivity. This suggests that aberrant TLR7-TRAF5 signaling causes self-reactive B-cells in systemic lupus erythematosus. TRAF5 is one of a group of genes related to cellular aging and senescence that decreases in expression as people age [28]. The single nucleotide polymorphisms (SNPs) of TRAF6 on Chromosome 11 are linked to autoimmune disorders, including rheumatoid arthritis (RA), systemic lupus erythematosus (SLE), and idiopathic inflammatory myopathy [29,30]. A single nucleotide polymorphism (SNP) in the TRAF6 gene has been linked to an increased vulnerability to acute lung injury caused by sepsis [31]. The Rv0222 protein derived from *M. tuberculosis* has the ability to bind to TRAF6 and effectively hinder the activation of MAPK and NF-κB pathways, which are typically mediated by TRAF6. This demonstrates a technique employed by *M. tuberculosis* to evade the host’s immune response [32].

In their 2019 study, Swaidani et al., 2019, elucidated the critical function of TRAFs in regulating IL-17 cytokine signaling pathways, emphasizing their dual role in both activating and resolving these pathways. The authors argue that the dysregulation of TRAFs is a key factor in various disease pathologies, including chronic inflammation and autoimmunity, suggesting that a deeper understanding of TRAF dynamics could lead to novel therapeutic strategies [33]. This understanding sets the stage for investigating the implications of TRAF dynamics in infectious diseases, particularly COVID-19. 

Correspondingly, TRAFs and platelets can interact in both direct and indirect ways. Studies have demonstrated that TRAF3 has a suppressive effect on platelet activation and thrombosis, whereas TRAF2 facilitates platelet activation, which is dependent on CD40L [34]. Thus, tumor necrosis factor receptor-associated factors (TRAFs) have essential functions in regulating platelet activity [34]. TRAF3 also plays a significant role in boosting the body’s natural defense against influenza A virus infection by strengthening the antiviral responses through the type-I IFN signaling pathway. This emphasizes the critical importance of TRAF3 in the immune system [35]. Platelet signaling pathways that connect inflammatory and thrombotic circuits have a crucial impact on thrombotic events associated with atherosclerosis [20]. The presence of pro-inflammatory receptors and their associated TRAFs, especially TRAF1 and TRAF5, influences platelet functioning and hemostasis, often exerting contrasting effects. The correlation between inflammatory and platelet-specific signaling offers a possible rationale for the increased occurrence of blood clotting difficulties in chronic inflammatory diseases [36]. TRAF6 signaling in dendritic cells enhances immunity by inducing Th1 and Th17 responses, protecting against infectious colitis caused by C. rodentium, and showcasing its crucial role in immune defense mechanisms [37]. TRAF7 negatively regulates the RLR signaling pathway by promoting K48-linked ubiquitination of TBK1, indicating TRAF’s role in modulating immunity through regulating key signaling molecules [38]. Studies have demonstrated that TRAF5 provides protection against cardiac ischemia reperfusion injury through AKT T signaling, highlighting the significant function of TRAF5 in inflammation and apoptosis [39]. In spite of the fact that it may appear to be controversial, the regulation of these genes is significantly influenced by the conditions and behaves differently depending on the disease that is being discussed.

Thrombotic complications, such as deep vein thrombosis (DVT) and pulmonary embolism (PE), are hallmarks of severe COVID-19. Platelets contribute to these complications through their interactions with endothelial cells, leukocytes, and the coagulation cascade [2]. TRAF proteins may influence these processes by promoting the release of pro-thrombotic factors (e.g., P-selectin, von Willebrand factor) from activated platelets, enhancing the formation of platelet–leukocyte aggregates, and modulating the balance between pro-inflammatory and anti-inflammatory signaling in platelets. Our observation that TRAF5 and 6 are upregulated in platelets from infected mice suggests that this molecule may play a role in mitigating thrombotic complications. The upregulation of TRAF5 and TRAF6 in platelets during SARS-CoV-2 infection significantly impacts immune cell responses by amplifying inflammatory signaling pathways. TRAF6, a key mediator of Toll-like receptor (TLR) and interleukin-1 receptor (IL-1R) signaling, activates NF-κB and MAPK pathways, leading to the production of pro-inflammatory cytokines such as IL-6, TNF-α, and IL-1β. This cytokine release enhances the recruitment and activation of immune cells, including neutrophils and macrophages, contributing to the cytokine storm observed in severe COVID-19. TRAF5, while less studied, modulates TNF receptor signaling and contributes to T-cell activation and inflammation. In platelets, their upregulation likely enhances platelet–leukocyte interactions and the release of immunomodulatory factors, further driving immune cell recruitment and tissue inflammation. Together, TRAF5 and TRAF6 serve as critical links between platelet activation and systemic immune dysregulation during viral infections. Further studies are needed to explore this possibility and determine whether targeting TRAF family signaling could provide therapeutic benefits for COVID-19.

The modulation of TRAF expression in platelets during SARS-CoV-2 infection has important implications for COVID-19 therapy and biomarker development. Additionally, targeting TRAF-mediated signaling pathways in platelets could provide new strategies for modulating immune responses to COVID-19. For instance, inhibitors of TRAF6 or TRAF2 may help mitigate cytokine storm, while agonists of TRAF3 could enhance antiviral IFN responses.

While our study provides novel insights into the role of platelet TRAF signaling in SARS-CoV-2 infection, several limitations must be acknowledged. The small sample size limits the statistical power of our findings. Future studies should include larger cohorts to validate these results. Additionally, the use of differential centrifugation for platelet isolation, while effective, may not fully exclude contaminating cells. Future work should incorporate flow cytometry to confirm platelet purity. Finally, the functional role of TRAF proteins in platelets during SARS-CoV-2 infection remains to be elucidated. Mechanistic studies using genetic or pharmacological approaches are needed to determine how TRAF signaling influences platelet activation, inflammation, and thrombosis in COVID-19.

In conclusion, our study highlights the potential role of TRAF proteins in platelet-mediated immune responses to SARS-CoV-2 infection. By modulating inflammatory signaling, cytokine production, and thrombotic processes, platelet TRAF molecules may contribute to the pathogenesis of COVID-19. These findings underscore the importance of platelets as immune modulators in viral infections and provide a foundation for future research into TRAF-targeted therapies for COVID-19 and other viral diseases.

## 5. Conclusions

Our findings indicate that the SARS-CoV-2 infection and the ChadOX1 vaccine may have an impact on the expression of the TRAF5 and TRAF6 genes, indicating that vaccination is still a viable strategy and demonstrating that these genes may play a significant part in the pathogenesis of the virus, which could lead to the development of novel treatment targets. The limits of our study encompass the reality that our cohort functioned as an initial impetus to highlight this research domain, and our findings do not yield a definitive conclusion about the matter. Conversely, they provide a foundation for subsequent investigations including larger cohorts and more sophisticated methodologies to elucidate the involvement of TRAFs in the pathophysiology of COVID-19 and in platelet function.

## Data Availability

The data that support the findings of this study are available from the corresponding author upon reasonable request.
